# Anti–SARS-CoV-2 Pharmacotherapies Among Nonhospitalized US Veterans, January 2022 to January 2023

**DOI:** 10.1001/jamanetworkopen.2023.31249

**Published:** 2023-08-31

**Authors:** Lei Yan, Elani Streja, Yuli Li, Nallakkandi Rajeevan, Mazhgan Rowneki, Kristin Berry, Denise M. Hynes, Francesca Cunningham, Grant D. Huang, Mihaela Aslan, George N. Ioannou, Kristina L. Bajema

**Affiliations:** 1 Veterans Affairs Cooperative Studies Program Clinical Epidemiology Research Center, Veterans Affairs Connecticut Healthcare System, West Haven, Connecticut; 2 Department of Biostatistics, Yale School of Public Health, New Haven, Connecticut; 3 Center of Innovation to Improve Veteran Involvement in Care, Veterans Affairs Portland Healthcare System, Portland, Oregon; 4 Research and Development, Veterans Affairs Puget Sound Health Care System, Seattle, Washington; 5 Health Management and Policy, School of Social and Behavioral Health Sciences, College of Public Health and Human Sciences; Health Data and Informatics Program, Center for Quantitative Life Sciences, Oregon State University, Corvallis; 6 Pharmacy Benefit Management Services, Veterans Affairs Center for Medication Safety, Hines, Illinois; 7 Office of Research and Development, Veterans Health Administration, Washington, District of Columbia; 8 Department of Medicine, Yale School of Medicine, New Haven, Connecticut; 9 Division of Gastroenterology, Veterans Affairs Puget Sound Healthcare System, Seattle, Washington; 10 Department of Medicine, University of Washington, Seattle; 11 Veterans Affairs Portland Health Care System, Portland, Oregon; 12 Division of Infectious Diseases, Department of Medicine, Oregon Health and Sciences University, Portland

## Abstract

**Importance:**

Several pharmacotherapies have been authorized to treat nonhospitalized persons with symptomatic COVID-19. Longitudinal information on the use of these therapies is needed.

**Objective:**

To analyze trends and factors associated with prescription of outpatient COVID-19 pharmacotherapies within the Veterans Health Administration (VHA).

**Design, Setting, and Participants:**

This cohort study evaluated nonhospitalized veterans in VHA care who tested positive for SARS-CoV-2 from January 2022 through January 2023 using VHA and linked Community Care and Medicare databases.

**Exposures:**

Demographic characteristics, underlying medical conditions, COVID-19 vaccination, and regional and local systems of care, including Veterans Integrated Services Networks (VISNs).

**Main Outcomes and Measures:**

Monthly receipt of any COVID-19 pharmacotherapy (nirmatrelvir-ritonavir, molnupiravir, sotrovimab, or bebtelovimab) was described. Multivariable logistic regression was used to identify factors independently associated with receipt of any vs no COVID-19 pharmacotherapy.

**Results:**

Among 285 710 veterans (median [IQR] age, 63.1 [49.9-73.7] years; 247 358 males [86.6%]; 28 444 Hispanic [10.0%]; 61 269 Black [21.4%] and 198 863 White [69.6%]) who tested positive for SARS-CoV-2 between January 2022 and January 2023, the proportion receiving any pharmacotherapy increased from 3285 of 102 343 veterans (3.2%) in January 2022 to 5180 of 21 688 veterans (23.9%) in August 2022. The proportion declined to 2194 of 10 551 veterans (20.8%) by January 2023. Across VISNs, the range in proportion of patients who tested positive who received nirmatrelvir-ritonavir or molnupiravir during January 2023 was 41 of 692 veterans (5.9%) to 106 of 494 veterans (21.4%) and 2.1% to 120 of 1074 veterans (11.1%), respectively. Veterans receiving any treatment were more likely to be older (adjusted odds ratio [aOR] for ages 65-74 vs 50-64 years, 1.18; 95% CI, 1.14-1.22; aOR for ages ≥75 vs 50-64 years, 1.19; 95% CI, 1.15-1.23) and have a higher Charlson Comorbidity Index score (aOR for CCI ≥6 vs 0, 1.52; 95% CI, 1.44-1.59). Compared with White veterans, Black veterans (aOR, 1.06; 95% CI, 1.02-1.09) were more likely to receive treatment, and compared with non-Hispanic veterans, Hispanic veterans (aOR 1.06; 95% CI, 1.01-1.11) were more likely to receive treatment.

**Conclusions And Relevance:**

This study found that prescription of outpatient COVID-19 pharmacotherapies in the VHA peaked in August 2022 and declined thereafter. There were large regional differences in patterns of nirmatrelvir-ritonavir and molnupiravir use.

## Key Points

**Question:**

How have anti–SARS-CoV-2 pharmacotherapies been used among nonhospitalized US Veterans in the Veterans Affairs health care system?

**Findings:**

In this cohort study of 285 710 outpatient US veterans who tested positive for SARS-CoV-2 from January 2022 through January 2023, the proportion receiving any pharmacotherapy increased from 3.2% in January 2022 to 23.9% in August 2022 and declined to 20.8% by January 2023. Black, Hispanic, and older veterans with a higher number of underlying conditions were more likely to receive treatment.

**Meaning:**

These results suggest the need for continued support of infrastructure and education to facilitate treatment for individuals at highest risk of progression to severe COVID-19.

## Teaser

This cohort study investigates monthly rates of receipt of COVID-19 pharmacotherapies among nonhospitalized patients in the VHA from January 2022 to January 2023.

## Introduction

Several SARS-CoV-2 antiviral agents are recommended in the US for the treatment of COVID-19 in nonhospitalized adults at risk for progressing to severe disease.^1^ These include ritonavir-boosted nirmatrelvir, remdesivir, and molnupiravir, which received US Food and Drug Administration (FDA) Emergency Use Authorization (EUA) between December 2021 and January 2022.^2^ Collectively, these pharmacotherapies have been demonstrated to aid clinical recovery and reduce the risk of hospitalization and death.^3,4,5,6,7,8^ After slow early uptake, 8.9 million courses of nirmatrelvir-ritonavir and 1.3 million courses of molnupiravir had been administered across the US by April 2023.^9,10,11,12^ Despite this, challenges that impede broader uptake among populations at increased risk remain, and information on use more than 1 year after authorization remains limited.

The Veterans Health Administration (VHA), operated by the US Department of Veterans Affairs (VA), is the largest integrated health care system in the US, serving more than 9 million enrolled veterans each year at 171 medical centers and 1113 outpatient sites of care.^13^ COVID-19 pharmacotherapies under EUA are allocated across VHA pharmacies through a national distribution system coordinated by the Pharmacy Benefits Management Services (PBM). This system provides an opportunity to examine how these therapies have been allocated to patients infected with SARS-CoV-2 over time.^9^ We sought to describe trends and factors associated with prescription of COVID-19 pharmacotherapies from January 2022 through January 2023, focusing on nirmatrelvir-ritonavir, molnupiravir, and historically available neutralizing monoclonal antibodies prescribed during this period.

## Methods

This cohort study was approved by the VA Central Institutional Review Board, which determined that patient consent was not required. We followed the Strengthening the Reporting of Observational Studies in Epidemiology (STROBE) reporting guideline.

### Data Sources

We used the VA COVID-19 Shared Data Resource, provisioned by the VA Informatics and Computing Infrastructure, which integrates multiple data sources to provide patient-level COVID-19–related information on VA enrollees.^14^ Positive SARS-CoV-2 tests among VA enrollees are identified by the VA National Surveillance Tool and provided to the COVID-19 Shared Data Resource to support national VA research and operations needs.^15^ The National Surveillance Tool identifies VA enrollees with a laboratory-confirmed positive SARS-CoV-2 nucleic acid amplification or antigen test performed within the VHA or evidence of testing positive outside of the VHA and documented in VHA clinical records. Information on non-VHA testing is obtained through recordings in templated notes yielding structured data or by natural language processing of the electronic health record (EHR), which is confirmed by manual EHR review.

COVID-19 pharmacotherapies were ascertained using 3 sources: (1) the VA Corporate Data Warehouse (CDW), a linked database of VHA EHRs that contains prescription records from inpatient and outpatient care; (2) Medicare claims data from the Centers for Medicare & Medicaid Services (CMS) provided by the VA Information Resource Center, which includes claims for veterans who used Medicare; and (3) COVID-19 monoclonal antibody claims data from the VA Community Care program, which coordinates and reimburses local care provided outside the VHA. For this analysis, CMS-Medicare and VA Community Care data were available through September 30, 2022. We also used the CDW to obtain patient-level demographic, clinical, and administrative data. Hospitalization data were obtained from CDW and CMS-Medicare.

### Study Population and Baseline Characteristics

We identified veterans aged 18 years or older with a first positive SARS-CoV-2 test between January 1, 2022, and January 31, 2023. The study population was limited to VA enrollees who had at least 1 VHA primary care outpatient encounter during the 18 months before the positive SARS-CoV-2 test and were not hospitalized on or within 7 days prior to the positive test.^9^

Using the date of the positive SARS-CoV-2 test as the index date, we ascertained baseline demographic characteristics, including race and ethnicity (associated with COVID-19 care) as reported in the VA EHR and enrollment records. Race and ethnicity were self-reported. Race options include American Indian or Alaska Native, Asian, Black or African American, Native Hawaiian or Other Pacific Islander, White, and unknown. Ethnicity options include Hispanic or Latino, not Hispanic or Latino, and unknown. American Indian or Alaska Native, Asian, and Native Hawaiian or Other Pacific Islander were aggregated into other race due to low population numbers; other race included self-identification as other or more than 1 race. Race and ethnicity were assessed because they are associated with levels of COVID-19 care. Additional demographic information included VHA facility and Veterans Integrated Service Network (VISN, the 18 regional systems of care)^16^ associated with the SARS-CoV-2 test and rurality of residence based on the Rural-Urban Commuting Areas system.^17^ We also determined smoking status, alcohol or substance use disorder, Charlson Comorbidity Index (CCI) score, COVID-19 vaccination status, and underlying medical conditions, including immunocompromised status, as previously described.^8,9^

### COVID-19 Pharmacotherapies

We identified receipt of nirmatrelvir-ritonavir, molnupiravir, and 2 anti–SARS-CoV-2 monoclonal antibodies in use during the study period (sotrovimab and bebtelovimab, designated as the monoclonal antibody group), as captured by VA CDW prescriptions, CMS-Medicare, and VA Community Care claims. Due to circulation of Omicron variants with reduced sensitivity to monoclonal antibodies, FDA authorization for sotrovimab was removed in April 2022 and authorization for bebtelovimab was removed in November 2022.^2^ By January 2022, use of bamlanivimab and etesevimab, as well as casirivimab and imdevimab, was already very limited due to reduced activity against Omicron variants; we therefore did not include these in the monoclonal antibody group.^18^ Although remdesivir was authorized by the FDA for the treatment of COVID-19 in nonhospitalized patients in January 2022,^19^ the number of individuals who received outpatient remdesivir was small and not readily distinguished from inpatient remdesivir; thus, we did not include this as a separate treatment group. Eligible veterans were assigned to a treatment group based on the first treatment received within 7 days before or after their first positive SARS-CoV-2 test. Individuals who did not receive any outpatient COVID-19 pharmacotherapy within 7 days before or after their test were assigned to the no treatment group.

### Statistical Analysis

Among patients who tested positive, we calculated the proportion prescribed each COVID-19 pharmacotherapy between January 2022 and January 2023 by month and according to VISN, VA facility, and demographic and clinical characteristics. To investigate factors associated with receipt of any treatment vs no treatment, we estimated unadjusted and adjusted odds ratios (aORs) with 95% CIs with binomial logistic regression models using data from April 2022 to January 2023, when the relative proportion of veterans who tested positive receiving outpatient COVID-19 pharmacotherapies had stabilized. Models were adjusted for age, sex, race, ethnicity, VISN, and CCI. To avoid overadjustment, we did not include CCI when evaluating individual underlying conditions. Final models used complete data for all included covariates. Analyses were conducted using R statistical software version 4.1.2 (R Project for Statistical Computing).

## Results

### Patient Characteristics

**Figure 1.**
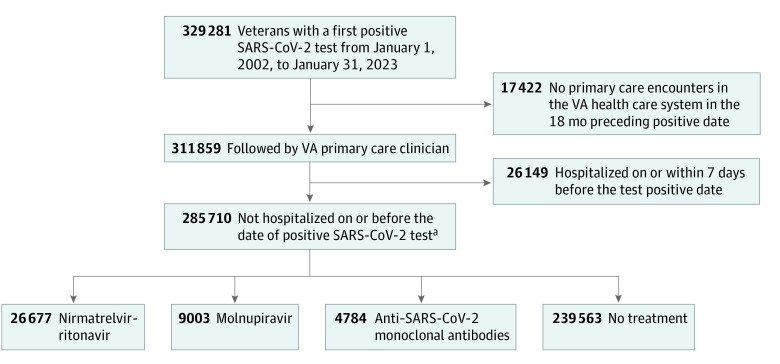
Study Flowchart US Department of Veterans Affairs (VA) enrollees with a first positive SARS-CoV-2 test from January 1, 2022, to January 31, 2023, included in the study are presented. ^a^A total of 5683 veterans who received remdesivir, bamlanivimab-etesevimab, and casirivimab-imdevimab were excluded from the treatment and no treatment groups.

**Figure 2.**
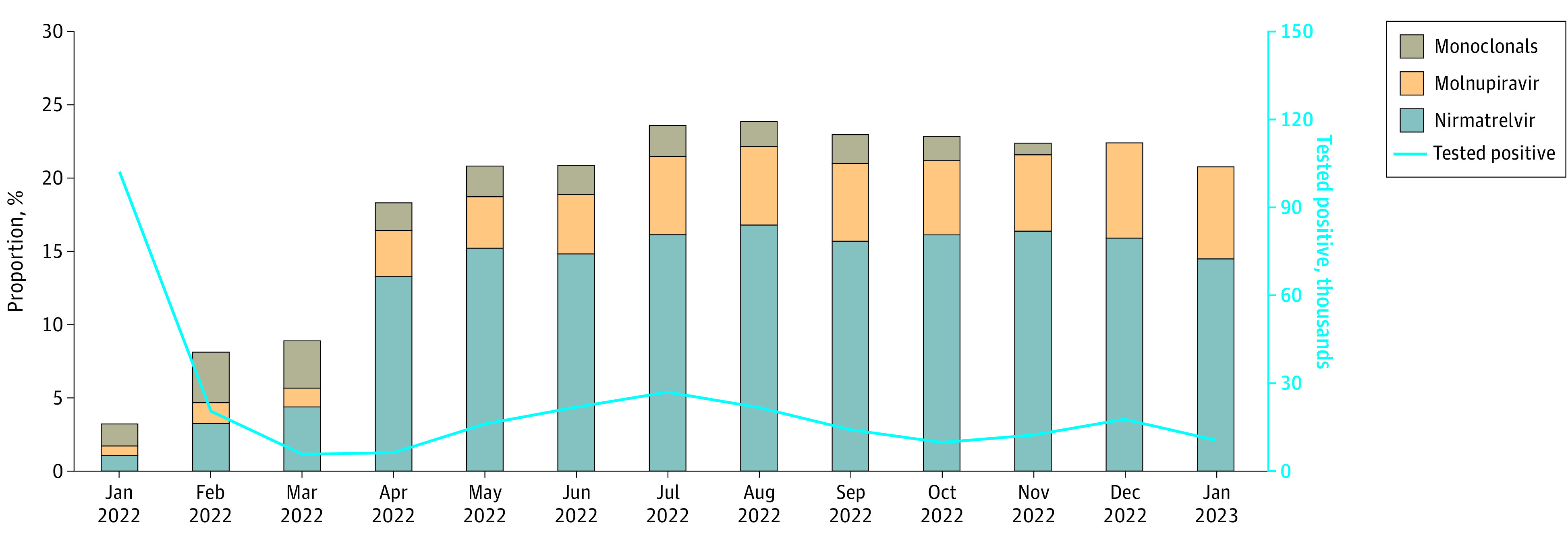
Distribution of Pharmacotherapies COVID-19 pharmacotherapies administered among patients who tested positive for SARS-CoV-2 in the Veterans Health Administration January 2022 to January 2023 are presented.

Between January 2022 and January 2023, 285 710 VA enrollees (median [IQR] age 63.1 [49.9-73.7] years; 247 358 males [86.6%]; 28 444 Hispanic [10.0%]; 61 269 Black [21.4%] and 198 863 White [69.6%]) tested positive for SARS-CoV-2 and fulfilled study inclusion criteria (Figure 1; Table 1).^18^ During January 2022, more than 102 343 veterans tested positive for SARS-CoV-2; this decreased to 20 450 the following month and remained relatively stable thereafter (Figure 2). Of all persons who tested positive, 26 677 individuals (9.3%) received nirmatrelvir-ritonavir, 9003 individuals (3.2%) received molnupiravir, 4784 individuals (1.7%) received monoclonal antibodies (sotrovimab or bebtelovimab), and 239 563 individuals (83.8%) did not receive treatment. The remaining 5683 individuals (2.0%) received other COVID-19 pharmacotherapies (5058 individuals [1.8%] received remdesivir, and 638 individuals [0.3%] received other monoclonal antibodies) and were not included in the any treatment or no treatment groups. Most pharmacotherapies were identified from the VA CDW (18 051 of 18 735 nirmatrelvir-ritonavir prescriptions [96.3%], 5837 of 6050 molnupiravir prescriptions [96.5%], and 3591 of 4527 monoclonal antibody treatments [79.3%]) (eTables 1 and 2 in Supplement 1). CMS-Medicare data contributed to 684 nirmatrelvir-ritonavir (3.7%) and 213 molnupiravir (3.5%) prescriptions and 420 monoclonal antibody treatments (9.3%). The remaining 516 monoclonal antibody treatments (11.4%) were from VA Community Care data. With regard to treatment rate, the CMS-Medicare and VA Community Care data contributed to 1833 additional treatments between January 2022 and September 2022, accounting for 0.8% of 23 5420 SARS-CoV-2 infections.

**Table 1.** Characteristics of Veterans

Characteristic	Veterans, No. (%)				
	All (N = 285 710)	No treatment (n = 239 563)	Nirmatrelvir-ritonavir (n = 26 677)	Molnupiravir (n = 9003)	Monoclonal antibodies (n = 4784)^a^
Age, y					
Median (IQR), y	63.1 (49.9-73.7)	61.7 (47.9-73.1)	67.0 (56.0-74.9)	71.8 (62.2-76.6)	72.4 (63.2-76.7)
Group					
18-49	71 872 (25.2)	66 294 (27.7)	4124 (15.5)	713 (7.9)	401 (8.4)
50-64	82 862 (29.0)	70 878 (29.6)	7779 (29.2)	2142 (23.8)	965 (20.2)
65-74	73 234 (25.6)	58 175 (24.3)	8241 (30.9)	3094 (34.4)	1762 (36.8)
≥75	57 737 (20.2)	44 210 (18.5)	6533 (24.5)	3054 (33.9)	1656 (34.6)
Sex					
Male	247 358 (86.6)	206 125 (86.0)	23 329 (87.4)	8222 (91.3)	4367 (91.3)
Female	38 352 (13.4)	33 438 (14.0)	3348 (12.6)	781 (8.7)	417 (8.7)
Race^b^					
Black	61 269 (21.4)	51 537 (21.5)	6045 (22.7)	1773 (19.7)	786 (16.4)
White	198 863 (69.6)	165 499 (69.1)	18 615 (69.8)	6742 (74.9)	3747 (78.3)
Other	13 548 (4.7)	11 664 (4.9)	1192 (4.5)	325 (3.6)	162 (3.4)
Missing	12 024 (4.2)	10 856 (4.5)	825 (3.1)	163 (1.8)	89 (1.9)
Hispanic ethnicity					
Hispanic	28 444 (10.0)	24 495 (10.2)	2437 (9.1)	694 (7.7)	336 (7.0)
Missing	11 043 (3.9)	9419 (3.9)	889 (3.3)	340 (3.8)	175 (3.7)
Rurality^c^					
Urban	216 544 (75.8)	181 425 (75.7)	20 881 (78.3)	6595 (73.3)	3352 (70.1)
Rural	69 161 (24.2)	58 132 (24.3)	5796 (21.7)	2408 (26.7)	1432 (29.9)
Missing	5 (<0.1)	6 (<0.1)	0	0	0
Region^d^					
West	70 913 (24.8)	59 562 (24.9)	6564 (24.6)	1975 (21.9)	1123 (23.5)
Midwest	51 481 (18.0)	42 326 (17.7)	5760 (21.6)	1372 (15.2)	1238 (25.9)
Northeast	45 885 (16.1)	36 820 (15.4)	5445 (20.4)	1579 (17.5)	1120 (23.4)
South	117 426 (41.1)	100 849 (42.1)	8908 (33.4)	4077 (45.3)	1303 (27.2)
Missing	5 (<0.1)	6 (<0.1)	0	0	0
VISN					
1	13 086 (4.6)	10 276 (4.3)	1800 (6.7)	535 (5.9)	206 (4.3)
2	12 042 (4.2)	9657 (4.0)	1374 (5.2)	539 (6.0)	240 (5.0)
4	11 492 (4.0)	9614 (4.0)	987 (3.7)	349 (3.9)	296 (6.2)
5	9265 (3.2)	7273 (3.0)	1284 (4.8)	156 (1.7)	378 (7.9)
6	21 638 (7.6)	18 258 (7.6)	1843 (6.9)	1089 (12.1)	213 (4.5)
7	17 680 (6.2)	15 376 (6.4)	1518 (5.7)	298 (3.3)	220 (4.6)
8	31 473 (11.0)	27 380 (11.4)	2049 (7.7)	1076 (12.0)	323 (6.8)
9	12 711 (4.4)	9718 (4.1)	1631 (6.1)	714 (7.9)	217 (4.5)
10	17 497 (6.1)	14 210 (5.9)	2084 (7.8)	551 (6.1)	374 (7.8)
12	10 870 (3.8)	8942 (3.7)	1274 (4.8)	178 (2.0)	277 (5.8)
15	10 353 (3.6)	8667 (3.6)	990 (3.7)	237 (2.6)	309 (6.5)
16	17 037 (6.0)	14 870 (6.2)	1014 (3.8)	584 (6.5)	249 (5.2)
17	16 887 (5.9)	15 247 (6.4)	853 (3.2)	316 (3.5)	81 (1.7)
19	13 618 (4.8)	11 857 (4.9)	775 (2.9)	610 (6.8)	182 (3.8)
20	10 795 (3.8)	9542 (4.0)	788 (3.0)	172 (1.9)	157 (3.3)
21	19 879 (7.0)	15 989 (6.7)	2333 (8.7)	564 (6.3)	297 (6.2)
22	26 621 (9.3)	22 174 (9.3)	2668 (10.0)	629 (7.0)	487 (10.2)
23	12 761 (4.5)	10 507 (4.4)	1412 (5.3)	406 (4.5)	278 (5.8)
Vaccination status^e^					
None	65 095 (22.8)	57 996 (24.2)	3809 (14.3)	1026 (11.4)	919 (19.2)
Partial	11 303 (4.0)	9443 (3.9)	840 (3.1)	268 (3.0)	313 (6.5)
Primary	90 383 (31.6)	78 648 (32.8)	6252 (23.4)	2141 (23.8)	1536 (32.1)
Booster	118 728 (41.6)	93 301 (38.9)	15 758 (59.1)	5566 (61.8)	2011 (42.0)
Other	196 (0.1)	169 (0.1)	18 (0.1)	2 (<0.1)	5 (0.1)
Smoking^f^					
Never	117 243 (41.0)	98 737 (41.2)	11 247 (42.2)	3460 (38.4)	1809 (37.8)
Former	111 801 (39.1)	91 837 (38.3)	11 126 (41.7)	4074 (45.3)	2242 (46.9)
Current	45 410 (15.9)	39 166 (16.3)	3532 (13.2)	1266 (14.1)	583 (12.2)
Missing	11 256 (3.9)	9823 (4.1)	772 (2.9)	203 (2.3)	150 (3.1)
Alcohol use disorder^f^					
No	225 682 (79.0)	187 718 (78.4)	21 638 (81.1)	7498 (83.3)	4101 (85.7)
Yes	60 017 (21.0)	51 834 (21.6)	5039 (18.9)	1504 (16.7)	683 (14.3)
Missing	11 (<0.1)	11 (<0.1)	0	1 (<0.1)	0
Nonalcohol substance use disorder^f^					
No	271 895 (95.2)	227 733 (95.1)	25 616 (96.0)	8565 (95.1)	4629 (96.8)
Yes	13 804 (4.8)	11 819 (4.9)	1061 (4.0)	437 (4.9)	155 (3.2)
Missing	11 (<0.1)	11 (<0.1)	0	1 (<0.1)	0
CCI score					
0	115 763 (40.5)	103 479 (43.2)	9035 (33.9)	1769 (19.7)	710 (14.8)
1	59 138 (20.7)	49 587 (20.7)	6103 (22.9)	1677 (18.6)	826 (17.3)
2	42 865 (15.0)	34 425 (14.4)	4908 (18.4)	1661 (18.5)	888 (18.6)
3	24 392 (8.5)	19 169 (8.0)	2703 (10.1)	1158 (12.9)	627 (13.1)
4-5	25 442 (8.9)	19 512 (8.1)	2332 (8.7)	1531 (17.0)	950 (19.9)
≥6	18 099 (6.3)	13 380 (5.6)	1596 (6.0)	1206 (13.4)	783 (16.4)
Underlying condition^f^					
Obesity (BMI ≥30)	133 557 (46.7)	112 133 (46.8)	12 747 (47.8)	4213 (46.8)	2247 (47.0)
Chronic kidney disease	38 381 (13.4)	30 011 (12.5)	3086 (11.6)	2256 (25.1)	1414 (29.6)
Diabetes	89 075 (31.2)	70 607 (29.5)	9346 (35.0)	4059 (45.1)	2302 (48.1)
Cancer	42 204 (14.8)	32 776 (13.7)	4655 (17.4)	1972 (21.9)	1318 (27.6)
Cardiovascular disease	91 153 (31.9)	71 345 (29.8)	9085 (34.1)	4819 (53.5)	2601 (54.4)
Chronic lung disease	84 140 (29.5)	66 811 (27.9)	8671 (32.5)	3910 (43.4)	2113 (44.2)
Dementia	10 823 (3.8)	8372 (3.5)	873 (3.3)	465 (5.2)	302 (6.3)
Cerebrovascular disease	15 809 (5.5)	12 276 (5.1)	1407 (5.3)	920 (10.2)	450 (9.4)
Chronic liver disease	25 712 (9.0)	20 865 (8.7)	2534 (9.5)	1005 (11.2)	564 (11.8)
Mental health condition^g^	134 505 (47.1)	114 032 (47.6)	11 609 (43.5)	4251 (47.2)	2044 (42.7)
Immunocompromised^h^	24 174 (8.5)	17 881 (7.5)	2653 (9.9)	1210 (13.4)	1204 (25.2)

Abbreviations: BMI, body mass index (calculated as weight in kilograms divided by height in meters squared); CCI, Charlson Comorbidity Index; VISN, Veterans Integrated Service Networks.

^a^ Includes sotrovimab, available January to April 2022, and bebtelovimab, available January to November 2022.

^b^ Other race includes Asian, American Indian or Alaska Native, and Native Hawaiian or Pacific Islander.

^c^ Based on rural-urban commuting area codes.

^d^ Based on VISNs, where West includes VISNs 19 to 22; Midwest includes VISNs 10, 12, 15, and 23; Northeast includes VSNs 1, 2, 4, and 5; and South includes VISNs 6 to 9 and 16 to 17.

^e^ Vaccination status is determined based on COVID-19 vaccine type, number of doses, and immunocompromised status, as previously described.^18^

^f^ Includes tobacco use, alcohol or substance use disorder, and underlying conditions documented in the 2 years prior to positive SARS-CoV-2 test.

^g^ Includes major depressive disorder, bipolar disorder, and schizophrenia.

^h^ Immunocompromised status is defined by recent prescription of immunosuppressive or cancer medications, having HIV with a CD4 cell count of 200 cells/mm^3^ or less, orhaving hematologic cancers, as previously described.^18^

Characteristics by treatment group are shown in Table 1. Median (IQR) ages were 61.7 (49.9-73.7) years in the no treatment group and 67.0 (56.0-74.9), 71.8 (62.2-76.6), and 72.4 (63.2-76.7) years in the nirmatrelvir-ritonavir, molnupiravir, and monoclonal antibody groups, respectively. There were 51 537 Black individuals (21.5%) in the no treatment group and 6045 Black individuals (22.7%), 1773 Black individuals (19.7%), and 786 Black individuals (16.4%) in the nirmatrelvir-ritonavir, molnupiravir, and monoclonal antibody groups, respectively. There were 24 495 Hispanic individuals (10.2%) in the no treatment group and 2437 Hispanic individuals (9.1%), 694 Hispanic individuals (7.7%), and 336 Hispanic individuals (7.0%) in the nirmatrelvir-ritonavir, molnupiravir, and monoclonal antibody groups, respectively. There were 165 499 White individuals (69.1%) in the no treatment group and 18 615 White individuals (69.8%), 6742 White individuals (74.9%), and 3747 White individuals (78.3%) in the nirmatrelvir-ritonavir, molnupiravir, and monoclonal antibody groups, respectively.

The proportion of veterans who completed primary or booster vaccination was highest in the molnupiravir group (7707 individuals [85.6%]), followed by nirmatrelvir-ritonavir (12 010 individuals [82.5%]), monoclonal antibody (3547 individuals [74.1%]), and no treatment (17 949 individuals [71.7%]) groups. The proportion of veterans with a CCI score of 4 or greater was highest in the monoclonal antibody group (1733 individuals [36.3%]), followed by molnupiravir (2737 individuals [30.4%]), nirmatrelvir-ritonavir (3928 individuals [14.7%]), and no treatment (32 892 individuals [13.7%]) groups. Monoclonal antibody and molnupiravir groups had the highest prevalence of cardiovascular disease (2601 individuals [54.4%] receiving monoclonal antibody, 4819 individuals [53.5%] receiving molnupiravir, 9085 individuals [34.1%] receiving nirmatrelvir-ritonavir, and 71 345 individuals [29.8] receiving no treatment), chronic kidney disease (2302 individuals [48.1%] receiving monoclonal antibody, 4059 individuals [45.1%] receiving molnupiravir, 9346 individuals [35.0%] receiving nirmatrelvir-ritonavir, and 70 607 individuals [29.5%] receiving no treatment), and immunocompromised status (1204 individuals [25.2%] receiving monoclonal antibody, 1210 individuals [13.4%] receiving molnupiravir, 2653 individuals [9.9%] receiving nirmatrelvir-ritonavir, and 17 881 individuals [7.5%] receiving no treatment).

### Temporal Trends in COVID-19 Treatments

The proportion of patients who tested positive for SARS-CoV-2 and received any treatment increased from 3285 of 102 343 patients (3.2%) in January 2022 to a peak of 5180 of 21 688 patients (23.9%) in August 2022 and was 2194 of 10 551 patients (20.8%) by January 2023 (Figure 2). The proportion of patients who tested positive who received nirmatrelvir-ritonavir increased from 1074 patients (1.0%) in January 2022 to 3649 patients (16.8%) in August 2022 and was 1531 patients (14.5%) by January 2023 (Figure 2). Reductions were most notable in the oldest (≥75 years) age group and among White and Hispanic veterans (eFigure 1 in Supplement 1). The proportion of patients who tested positive who received molnupiravir increased from 676 patients (0.7%) in January to 1167 patients (5.4%) in August and was 663 patients (6.3%) by January 2023. The proportion treated with monoclonal antibody was highest in February 2022 (706 of 20 450 patients [3.5%]) and gradually declined to 643 of 12 296 patients (0.8%) by November 2022. Monoclonal antibodies were no longer prescribed as of December 2022.

### Regional Patterns in COVID-19 Treatments

**Figure 3.**
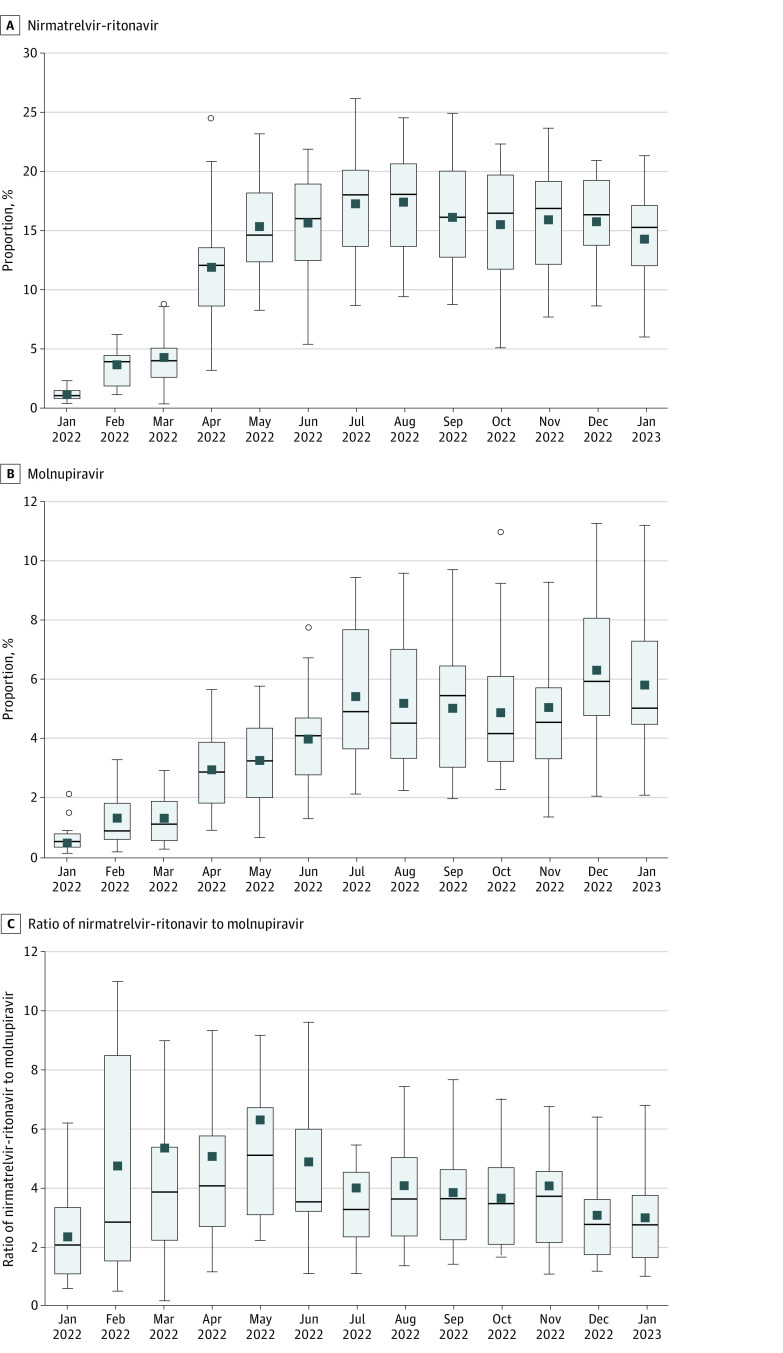
Distribution of Pharmacotherapies Across Veterans Integrated Services Network (VISN) COVID-19 pharmacotherapies across the 18 VISNs are presented by month. Boxes indicate IQRs; circles, outliers; horizontal lines, medians; squares, means; whiskers, 1.5 × the IQR. Outliers are shown in A and B but omitted in C.

There was substantial variation by VISN in the proportion of patients who tested positive who received nirmatrelvir-ritonavir and molnupiravir, which increased notably in April 2022 and persisted through the end of the study (Figure 3). By January 2023, the range in proportions was 41 of 692 patients (5.9%) at VISN 17 to 106 of 494 patients (21.4%) at VISN 9 in the nirmatrelvir-ritonavir group and 2.1% at VISN 20 to 120 of 1074 patients (11.1%) at VISN 6 in the molnupiravir group. The ratio of veterans receiving nirmatrelvir-ritonavir relative to molnupiravir during January 2023 varied across VISNs from 37 vs 36 veterans (1.0) at VISN 19 to 6.8 at VISN 20, suggesting that more nirmatrelvir-ritonavir was prescribed at the VISN level. There was also large variability in the proportions of veterans prescribed nirmatrelvir-ritonavir and molnupiravir across VA facilities (eFigure 2 in Supplement 1). Of 130 facilities that prescribed either oral antiviral, 12 facilities (9.2%) prescribed more molnupiravir compared with nirmatrelvir-ritonavir.

### Factors Associated With COVID-19 Treatment

Between April 2022 and January 2023, factors independently associated with higher likelihood of COVID-19 treatment were older age (aOR for ages 65-74 vs 50-64 years, 1.18; 95% CI, 1.14-1.22 ; aOR for ages ≥75 vs 50-64 years, 1.19; 95% CI, 1.15-1.23), Black vs White race (aOR, 1.06; 95% CI, 1.02-1.09), Hispanic ethnicity (aOR, 1.06; 95% CI, 1.01-1.11), higher CCI score (aOR for 4-5 vs 0, 1.39; 95% CI, 1.33-1.45; aOR for ≥6 vs 0, 1.52; 95% CI, 1.44-1.59), and receipt of vaccination vs no vaccination (aOR for primary vaccination, 1.25; 95% CI, 1.19-1.30; aOR for booster vaccination, 1.47; 95% CI, 1.42-1.53). Factors independently associated with lower likelihood of treatment included rural vs urban residence (aOR, 0.84; 95% CI, 0.82-0.87), current smoking (aOR, 0.84; 95% CI, 0.81-0.88), alcohol use disorder (aOR, 0.90; 95% CI, 0.87-0.92), and substance use disorder (aOR, 0.92; 95% CI, 0.86-0.98). Compared with veterans in VISN 1, those enrolled in all other VISNs except VISN 9 were less likely to receive treatment (Table 2).^18^

**Table 2.** Factors Associated With Receipt of Any COVID-19 Pharmacotherapy, April 2022 to January 2023

Factor	Patients receiving treatment, No./No. testing positive (%) (N = 15 4207)^a^	OR (95% CI)	
		Unadjusted	Adjusted^b^
Age, y			
18-49	4552/31 861 (14.3)	0.60 (0.57-0.62)	0.66 (0.63-0.68)
50-64	9569/43 741 (21.9)	1 [Reference]	1 [Reference]
65-74	11 128/42 223 (26.4)	1.28 (1.24-1.32)	1.18 (1.14-1.22)
≥75	9757/36 382 (26.8)	1.31 (1.27-1.35)	1.19 (1.15-1.23)
Sex			
Female	4036/20 219 (20.0)	0.83 (0.80-0.86)	1.08 (1.04-1.12)
Male	30 970/133 988 (23.1)	1 [Reference]	1 [Reference]
Race			
Black	7876/34 342 (22.9)	0.99 (0.96-1.02)	1.06 (1.02-1.09)
White	24 656/106 799 (23.1)	1 [Reference]	1 [Reference]
Other^c^	1502/7145 (21.0)	0.89 (0.84-0.94)	0.97 (0.91-1.03)
Ethnicity			
Hispanic	3084/15 615 (19.8)	0.82 (0.79-0.86)	1.06 (1.01-1.11)
Not Hispanic	31 922/138 592 (23.0)	1 [Reference]NA	1 [Reference]NA
VISN			
1	2259/7621 (29.6)	1 [Reference]	1 [Reference]
2	1928/7550 (25.5)	0.81 (0.76-0.87)	0.81 (0.75-0.87)
4	1376/6565 (21.0)	0.63 (0.58-0.68)	0.62 (0.57-0.67)
5	1501/5493 (27.3)	0.89 (0.83-0.96)	0.89 (0.82-0.96)
6	2800/11 641 (24.1)	0.75 (0.70-0.80)	0.76 (0.71-0.82)
7	1793/9122 (19.7)	0.58 (0.54-0.62)	0.59 (0.55-0.64)
8	3049/18 144 (16.8)	0.48 (0.45-0.51)	0.48 (0.45-0.51)
9	2100/6565 (32.0)	1.12 (1.04-1.20)	1.11 (1.03-1.20)
10	2592/9282 (27.9)	0.92 (0.86-0.98)	0.90 (0.84-0.96)
12	1552/6482 (23.9)	0.75 (0.69-0.81)	0.73 (0.68-0.79)
15	1247/5083 (24.5)	0.77 (0.71-0.84)	0.78 (0.72-0.85)
16	1624/8668 (18.7)	0.55 (0.51-0.59)	0.55 (0.51-0.59)
17	1022/7777 (13.1)	0.36 (0.33-0.39)	0.38 (0.35-0.41)
19	1252/6899 (18.1)	0.53 (0.49-0.57)	0.54 (0.50-0.59)
20	939/5300 (17.7)	0.51 (0.47-0.56)	0.53 (0.48-0.58)
21	2838/10 910 (26.0)	0.83 (0.78-0.89)	0.85 (0.79-0.91)
22	3429/14 142 (24.2)	0.76 (0.71-0.81)	0.80 (0.75-0.85)
23	1705/6963 (24.5)	0.77 (0.72-0.83)	0.77 (0.72-0.83)
CCI score			
0	10 297/58 292 (17.7)	1 [Reference]	1 [Reference]
1	7498/32 267 (23.2)	1.41 (1.36-1.46)	1.26 (1.21-1.30)
2	6393/24 534 (26.1)	1.64 (1.59-1.70)	1.37 (1.32-1.42)
3	3851/14 235 (27.1)	1.73 (1.66-1.80)	1.39 (1.33-1.45)
4-5	4009/14 666 (27.3)	1.75 (1.68-1.83)	1.39 (1.33-1.45)
≥6	2958/10 213 (29.0)	1.90 (1.81-1.99)	1.52 (1.44-1.59)
Rurality^d^			
Urban	27 223/118 403 (23.0)	1 [Reference]	1 [Reference]
Rural	7783/35 804 (21.7)	0.93 (0.90-0.96)	0.84 (0.82-0.87)
Smoking status			
Never	14 507/64 657 (22.4)	1 [Reference]	1 [Reference]
Former	14 973/61 656 (24.3)	1.11 (1.08-1.14)	0.98 (0.95-1.01)
Current	4600/22 992 (20.0)	0.86 (0.83-0.90)	0.84 (0.81-0.88)
Alcohol use disorder			
No	28 654/122 719 (23.3)	1 [Reference]	1 [Reference]
Yes	6352/31 488 (20.2)	0.83 (0.80-0.86)	0.90 (0.87-0.92)
Nonalcohol substance use disorder			
No	33 525/146 989 (22.8)	1 [Reference]	1 [Reference]
Yes	1481/7218 (20.5)	0.87 (0.82-0.93)	0.92 (0.86-0.98)
Vaccination status^e^			
None	4360/26 777 (16.3)	1 [Reference]	1 [Reference]
Partial	1037/4908 (21.1)	1.38 (1.28-1.49)	1.24 (1.14-1.34)
Primary	7834/38 081 (20.6)	1.33 (1.28-1.39)	1.25 (1.19-1.30)
Booster	21754/84 337 (25.8)	1.79 (1.72-1.85)	1.47 (1.42-1.53)
Other	21/104 (20.2)	1.30 (0.81-2.10)	1.13 (0.68-1.88)
Cancer			
No	28 267/129 308 (21.9)	1 [Reference]	1 [Reference]
Yes	6739/24 899 (27.1)	1.33 (1.29-1.37)	1.12 (1.09-1.16)
Cardiovascular disease			
No	21 008/101 631 (20.7)	1 [Reference]	1 [Reference]
Yes	13 998/52 576 (26.6)	1.39 (1.36-1.43)	1.16 (1.13-1.19)
Chronic kidney disease			
No	29417/132297 (22.2)	1 [Reference]	1 [Reference]
Yes	5589/21 910 (25.5)	1.20 (1.16-1.24)	1.02 (0.98-1.05)
Chronic lung disease			
No	22 503/106 892 (21.1)	1 [Reference]	1 [Reference]
Yes	12 503/47 315 (26.4)	1.35 (1.31-1.38)	1.22 (1.19-1.25)
Diabetes			
No	21 637/103 912 (20.8)	1 [Reference]	1 [Reference]
Yes	13 369/50 295 (26.6)	1.38 (1.34-1.41)	1.19 (1.16-1.23)
Immunocompromised^f^			
No	31 020/140 837 (22.0)	1 [Reference]	1 [Reference]
Yes	3986/13 370 (29.8)	1.50 (1.45-1.56)	1.35 (1.30-1.41)
Mental health condition^g^			
No	19 488/83 207 (23.4)	1 [Reference]	1 [Reference]
Yes	15 518/71 000 (21.9)	0.91 (0.89-0.94)	1.05 (1.02-1.08)
Obese (BMI ≥30)			
No	18 606/84 519 (22.0)	1 [Reference]	1 [Reference]
Yes	16 400/69 688 (23.5)	1.09 (1.06-1.12)	1.17 (1.14-1.20)

Abbreviations: BMI, body mass index (calculated as weight in kilograms divided by height in meters squared); CCI, Charlson Comorbidity Index; OR, odds ratio; VISN, Veterans Integrated Service Networks.

^a^ A total of 35 006 veterans who received nirmatrelvir, molnupiravir, and monoclonal antibody among 154 207 veterans testing positive for SARS-CoV-2 for April 2022 to January 2023 were included. Models were limited to veterans with complete data for all included covariates.

^b^ All models are adjusted for age, sex, race, ethnicity, VISN, and CCI. To avoid overadjustment, CCI was not included when evaluating individual underlying conditions.

^c^ Other race includes American Indian or Alaska Native, Asian, and Native Hawaiian or Pacific Islander.

^d^ Based on rural-urban commuting area codes.

^e^ Vaccination status is determined based on the COVID-19 vaccine type, number of doses, and immunocompromised status, as previously described.^18^

^f^ Immunocompromised status is defined by recent prescription of immunosuppressive or cancer medications, having HIV with a CD4 cell count of 200 cells/mm^3^ or less, or having hematologic cancers, as previously described.^18^

^g^ Includes major depressive disorder, bipolar disorder, and schizophrenia.

## Discussion

This cohort study found that among 285 710 nonhospitalized US veterans who tested positive for SARS-CoV-2 between January 2022 and January 2023, the proportion receiving any outpatient pharmacotherapy increased substantially from 3.2% to 23.9% between January and August 2022 and decreased to 20.8% by January 2023. Although nirmatrelvir-ritonavir remained the most prescribed treatment, the ratio of nirmatrelvir-ritonavir to molnupiravir prescribing decreased from May 2022 to January 2023. There were notable regional differences by VISN in the relative use of different pharmacotherapies. Older veterans with a higher burden of underlying conditions and Black and Hispanic individuals were more likely to receive treatment, whereas unvaccinated veterans and those living in rural areas were less likely to receive treatment.

Several factors may have been associated with the overall decline in the proportion of veterans receiving treatment after August 2022. The absence of available CMS-Medicare or VA Community Care data after September 2022 may explain a part of the decline given that these data contributed an additional 0.8% to the monthly treatment rate between January and September 2022. A relative increase in asymptomatic infections or milder disease could have occurred in the setting of improved COVID-19 vaccination or previous infection.^20^ Furthermore, overall reductions in COVID-19–related hospitalizations and deaths, as well as relaxation of restrictions implemented during the pandemic may have been associated with changes in risk perception and care-seeking behavior.^21^ Changes in infrastructure supporting COVID-19 care may also have been associated with prescribing. Although trends in early uptake of COVID-19 pharmacotherapies within the VHA mirrored other settings, a more recent decline has not been widely reported in nonveteran populations.^10,22^

The overall small decline in treatment was largely associated with reduced nirmatrelvir-ritonavir dispensing, which remained the most prescribed pharmacotherapy. Possible reasons for this observation include clinician and patient concerns about COVID-19 rebound after completion of nirmatrelvir-ritonavir, as well as challenges in identifying and managing drug-drug interactions.^4^ In contrast, dispensing of molnupiravir increased during the study period, most notably after FDA authorization for monoclonal antibodies was withdrawn in the setting of reduced susceptibility of circulating Omicron variants. Patients receiving molnupiravir, similar to patients who had received monoclonal antibodies, were older and had a higher burden of underlying conditions compared with other groups and therefore may not have been eligible to receive nirmatrelvir-ritonavir due to kidney or drug-drug contraindications.^23^

Use of COVID-19 pharmacotherapies varied greatly across VISNs and VA facilities and was not limited by distribution of drug supplies to VHA pharmacies. Although nirmatrelvir-ritonavir was preferentially recommended over molnupiravir and had more robust evidence supporting its clinical effectiveness, a number of facilities prescribed more molnupiravir than nirmatrelvir-ritonavir.^1,3,5,6,22,24^ Altogether, these differences may be associated with regional and local variation in policy, infrastructure, education, and clinician preferences. For example, higher likelihood of treatment in the VA New England Healthcare System (VISN 1) compared with nearly all other VISNs may have been associated with the regional Test To Treat pilot program.

Our previous work showed that Black and Hispanic veterans who tested positive for SARS-CoV-2 during January and February 2022 were less likely to receive outpatient COVID-19 treatment.^9^ However, over an expanded period of time, Black and Hispanic veterans were more likely to receive treatment. This suggests important progress in improving outreach to these minority racial and ethnic groups. Moreover, compared with nonveteran populations, VHA populations often have less pronounced racial and ethnic disparities in COVID-19–related care.^11^

The association of older age and underlying conditions with receipt of COVID-19 treatment is consistent with evidence that these groups are at higher risk for severe COVID-19 outcomes.^25,26^ A treatment pattern consistent with higher risk was not observed among veterans who were unvaccinated, who were less likely to receive any pharmacotherapy. This may reflect differences in patient behaviors, with veterans who seek vaccination also more likely to pursue treatment for COVID-19. In contrast to our earlier findings, rural veterans were less likely to receive any pharmacotherapy, which may reflect challenges in meeting a higher volume of oral antiviral treatment demand over time.^9^ It is also possible that rural veterans were more likely to seek care outside of the VHA, and although we included data from VA Community Care and CMS-Medicare claims, we may still have underascertained prescribing in this group. Persons with alcohol and substance use disorders were also less likely to receive treatment, suggesting a need for targeted outreach to these groups at increased risk.

### Limitations

This study has several limitations. First, as previously described, we could not fully ascertain whether veterans were symptomatic at the time of testing positive and truly eligible for treatment under EUA criteria.^9^ Trends in prescribing may have been impacted by changes in the relative proportion of asymptomatic infections over time. Second, although we integrated multiple databases, including the VHA CDW, CMS-Medicare, and VA Community Care claims to determine receipt of COVID-19 pharmacotherapies, we may not have accounted for all treatments provided outside of the VHA, particularly among veterans younger than age 65 years not enrolled in Medicare. In addition, data from CMS-Medicare and VA Community Care were available only through September 2022. However, the relative contributions to ascertainment of COVID-19 treatments between January and September 2022 were small and fairly stable. Third, we did not capture positive SARS-CoV-2 laboratory tests performed outside the VHA or results of self-testing not documented in VHA clinical records. Given our study eligibility criteria, this may have impacted measurement of additional infections and COVID-19 treatments. Fourth, we were not able to enumerate the number of veterans who were offered but declined treatment. Fifth, given differences in veteran demographic and clinical characteristics compared with the general US population and differences in care delivery between VHA and non-VHA systems, findings from this study may not be generalizable to other groups.

## Conclusions

In this nationwide cohort study of US veterans in VHA care who tested positive for SARS-CoV-2 between January 2022 and January 2023, prescription of outpatient COVID-19 pharmacotherapies steadily increased until August 2022 and declined slightly thereafter. This occurred in the setting of a relative increase in molnupiravir use, a reduction in nirmatrelvir-ritonavir use, and cessation of monoclonal antibody administration after removal of FDA authorization. Demographic, clinical, and geographic differences in systems of care were associated with the likelihood of receiving treatment. These results highlight important progress in reaching certain groups, including Black and Hispanic veterans. They also suggest the need for continued support of education and infrastructure, including staffing, to facilitate treatment for individuals at highest risk of progression to severe COVID-19. Future research should focus on evaluating the relative benefits and risks of available outpatient COVID-19 pharmacotherapies across different patient populations.

## Notes

Supplement 1. **eTable 1.** Distribution of COVID-19 Pharmacotherapies by Month and Data Source **eTable 2.** Distribution of COVID-19 Pharmacotherapies by Data Source **eFigure 1.** Monthly Receipt of Nirmatrelvir-Ritonavir and Molnupiravir by Demographic and Clinical Characteristic **eFigure 2.** Distribution of COVID-19 Pharmacotherapies by Veterans Health Administration Facility

Supplement 2.
Data Sharing Statement
