# Incorrect Date in Figure 1

**DOI:** 10.1001/jamanetworkopen.2023.40494

**Published:** 2023-10-16

In the Original Investigation titled “Anti–SARS-CoV-2 Pharmacotherapies Among Nonhospitalized US Veterans, January 2022 to January 2023,”^1^ published August 31, 2023, an incorrect beginning date for the study period was fixed in Figure 1; the date should have been January 1, 2022.^1^
