# Supplementary material for: Anti–SARS-CoV-2 Pharmacotherapies Among Nonhospitalized US Veterans, January 2022 to January 2023
Source: JAMA Netw Open. 2023 Aug 31;6(8):e2331249. doi: 10.1001/jamanetworkopen.2023.31249 (PMC10472184; doi:10.1001/jamanetworkopen.2023.31249)
Supplement: Supplement 1. — eTable 1. Distribution of COVID-19 Pharmacotherapies by Month and Data Source eTable 2. Distribution of COVID-19 Pharmacotherapies by Data Source eFigure 1. Monthly Receipt of Nirmatrelvir-Ritonavir and Molnupiravir by Demographic and Clinical Characteristic eFigure 2. Distribution of COVID-19 Pharmacotherapies by Veterans Health Administration Facility [file jamanetwopen-e2331249-s001.pdf]

Supplemental Online Content

Yan L, Streja E, Li Y, et al. Anti–SARS-CoV-2 pharmacotherapies among nonhospitalized US veterans, January 2022 to January 2023. *JAMA Netw Open*. 2023;6(8):e2331249. doi:10.1001/jamanetworkopen.2023.31249

eTable 1. Distribution of COVID-19 Pharmacotherapies by Month and Data Source

eTable 2. Distribution of COVID-19 Pharmacotherapies by Data Source

eFigure 1. Monthly Receipt of Nirmatrelvir-Ritonavir and Molnupiravir by Demographic and Clinical Characteristic

eFigure 2. Distribution of COVID-19 Pharmacotherapies by Veterans Health Administration Facility

This supplemental material has been provided by the authors to give readers additional information about their work.

© 2023 Yan L et al. *JAMA Network Open.*

Supplemental Table 1. Distribution of COVID-19 pharmacotherapies by month and data source

		Jan-2022	Feb	Mar	Apr	May	Jun	Jul	Aug	Sept	Oct	Nov	Dec	Jan-2023	Total
SARS-CoV-2 Infection		102,343	20,450	5,724	6,249	16,089	21,804	26,985	21,688	14,088	9,727	12,296	17,716	10,551	285,710
Prescriptions In VA CDW	Nirmatrelvir-ritonavir	1062	654	244	812	2359	3111	4210	3507	2092	1524	2017	2822	1531	25,945
	Molnupiravir	652	280	74	192	550	860	1398	1122	709	480	643	1153	663	8,776
	Monoclonals	1200	556	141	89	275	350	470	284	226	148	84	0	0	3,823
Additional[a] Prescriptions in CMS[b]	Nirmatrelvir-ritonavir or Molnupiravir	36	23	<11*	24	110	153	197	186	161	-	-	-	-	897
	Monoclonals	158	74	22	19	24	35	34	31	23	-	-	-	-	420
Additional[a] Prescriptions In Community Care[b]	Nirmatrelvir-ritonavir or Molnupiravir	0	0	0	0	0	0	0	0	0	-	-	-	-	0
	Monoclonals	177	76	22	10	38	47	67	49	30	-	-	-	-	516

Abbreviations: CMS, Centers for Medicare & Medicaid Services; VA CDW, Veterans Affairs Corporate Data Warehouse.

[a] These include prescriptions not already captured in VA CDW.
[b] Prescription data from CMS and Community care are only available through September 2022.
* Data from CMS were combined and masked in accordance with CMS cell size suppression policy (https://resdac.org/articles/cms-cell-size-suppression-policy).

© 2023 Yan L et al. *JAMA Network Open.*

Supplemental Table 2. Distribution of COVID-19 pharmacotherapies by data source[a]

	Jan-22	Feb-22	March-22	April-22	May-22	June-22	July-22	Aug-22	Sep-22
**Nirmatrelvir-ritonavir, %**									
VA CDW	99	98	97	98	96	96	96	96	94
CMS	1	2	3	2	4	4	4	4	6
Community care	0	0	0	0	0	0	0	0	0
**Molnupiravir, %**									
VA CDW	96	97	100	97	97	97	97	96	95
CMS	4	3	0	3	3	3	3	4	5
Community care	0	0	0	0	0	0	0	0	0
**Monoclonal antibodies, %**									
VA CDW	78	79	76	75	82	81	82	78	81
CMS	10	10	12	16	7	8	6	9	8
Community care	12	11	12	8	11	11	12	13	11

Abbreviations: CMS, Centers for Medicare & Medicaid Services; VA CDW, Veterans Affairs Corporate Data Warehouse.

[a] These include prescriptions not already captured in VA CDW. Prescription data from CMS and Community care are only available through September 2022.

© 2023 Yan L et al. *JAMA Network Open.*

Supplemental Figure 1. Monthly receipt of nirmatrelvir-ritonavir and molnupiravir by demographic and clinical characteristics

A. Age

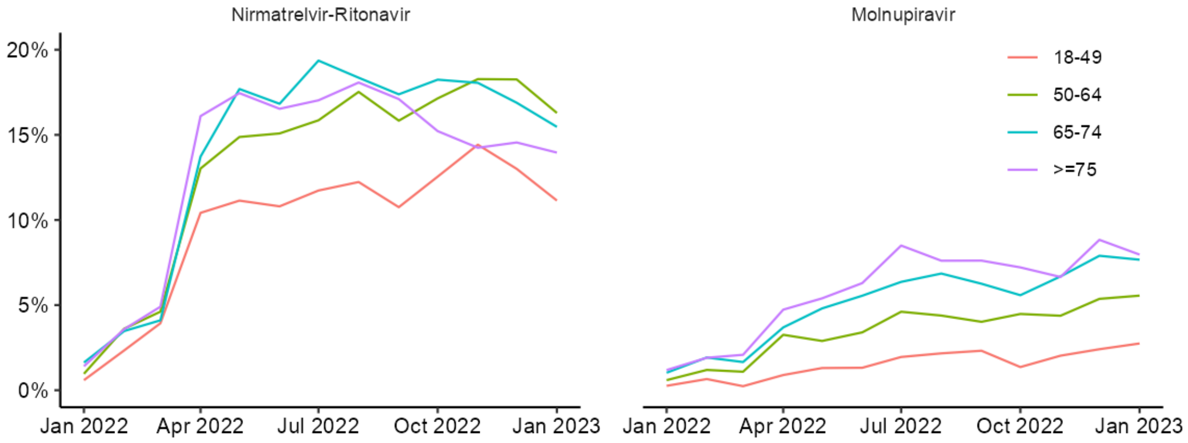

B. Race

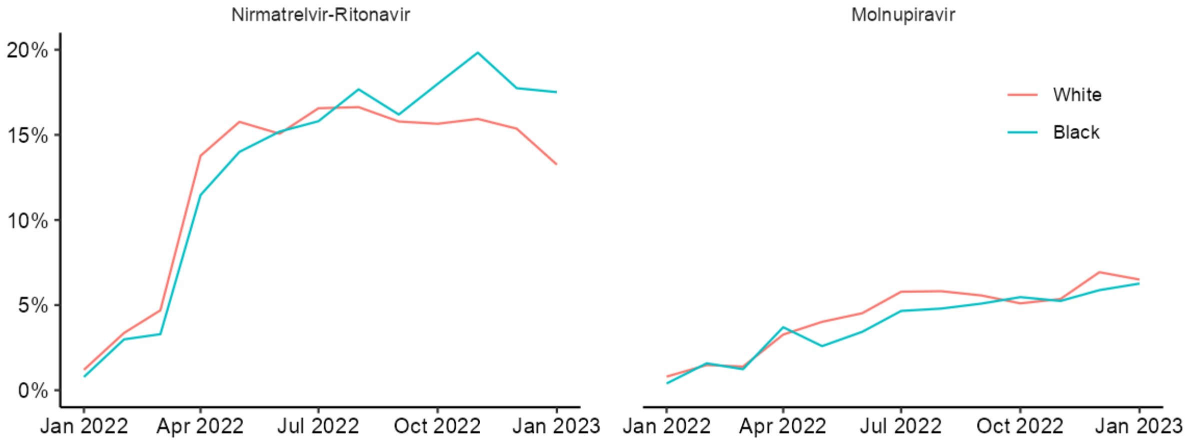

C. Ethnicity

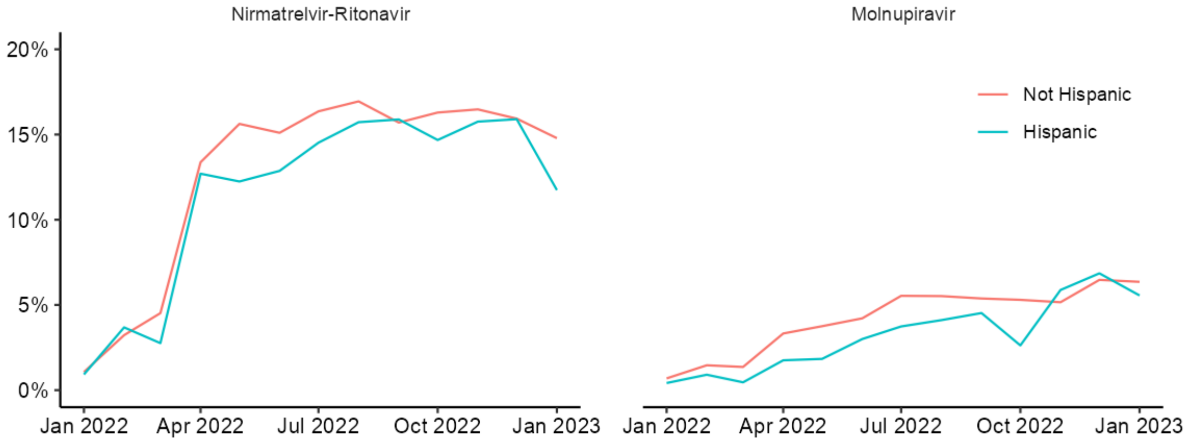

© 2023 Yan L et al. *JAMA Network Open.*

D. Vaccination status

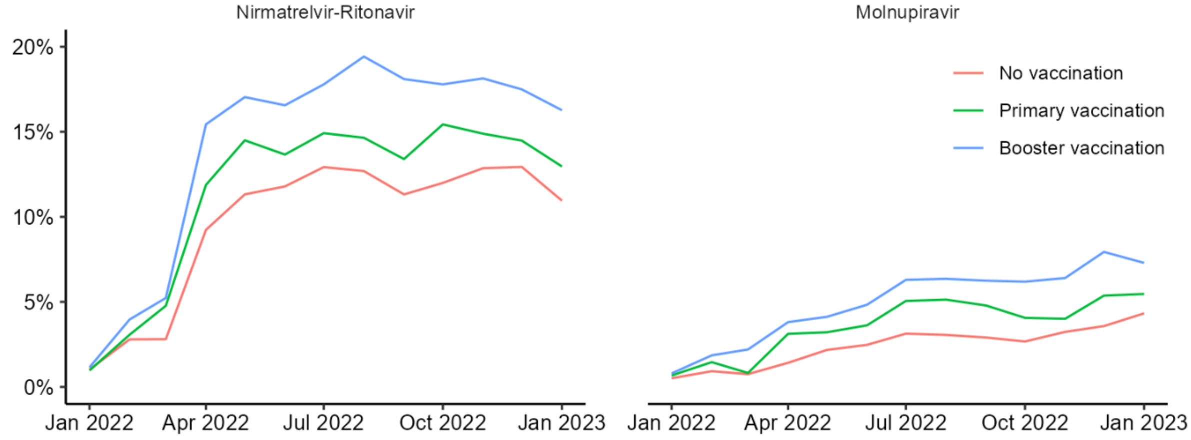

E. Charlson comorbidity index

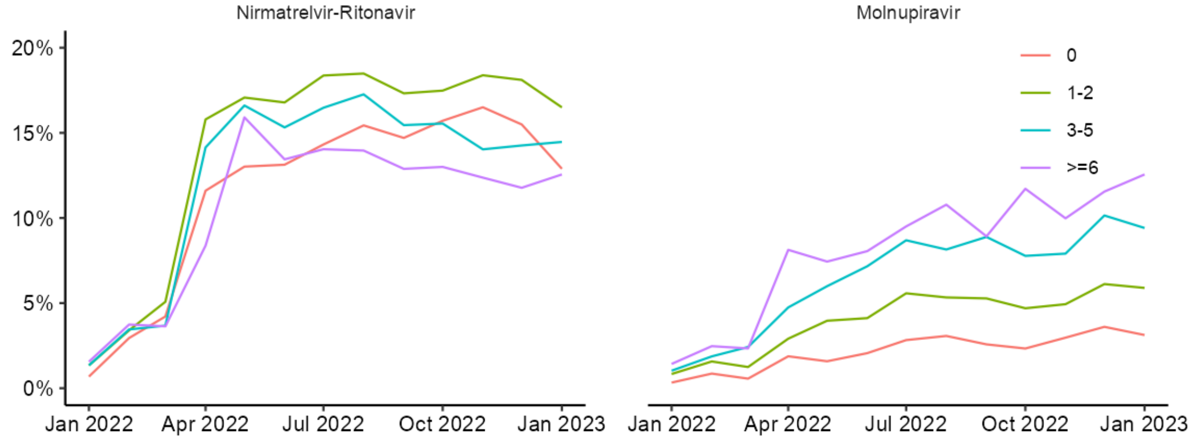

© 2023 Yan L et al. *JAMA Network Open.*

Supplemental Figure 2. Distribution of COVID-19 pharmacotherapies by Veterans Health Administration facility

A. Distribution of the proportions of persons receiving nirmatrelvir-ritonavir and molnupiravir across facilities, stratified by month

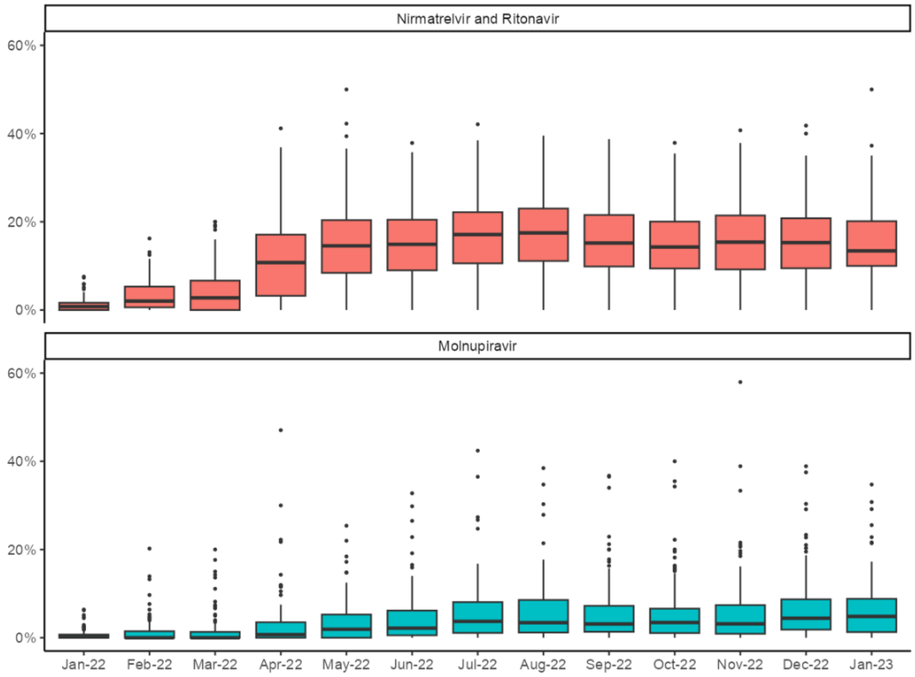

B. Distribution of the proportions of persons receiving nirmatrelvir-ritonavir and molnupiravir across facilities, stratified by facility complexity[a]

© 2023 Yan L et al. *JAMA Network Open.*

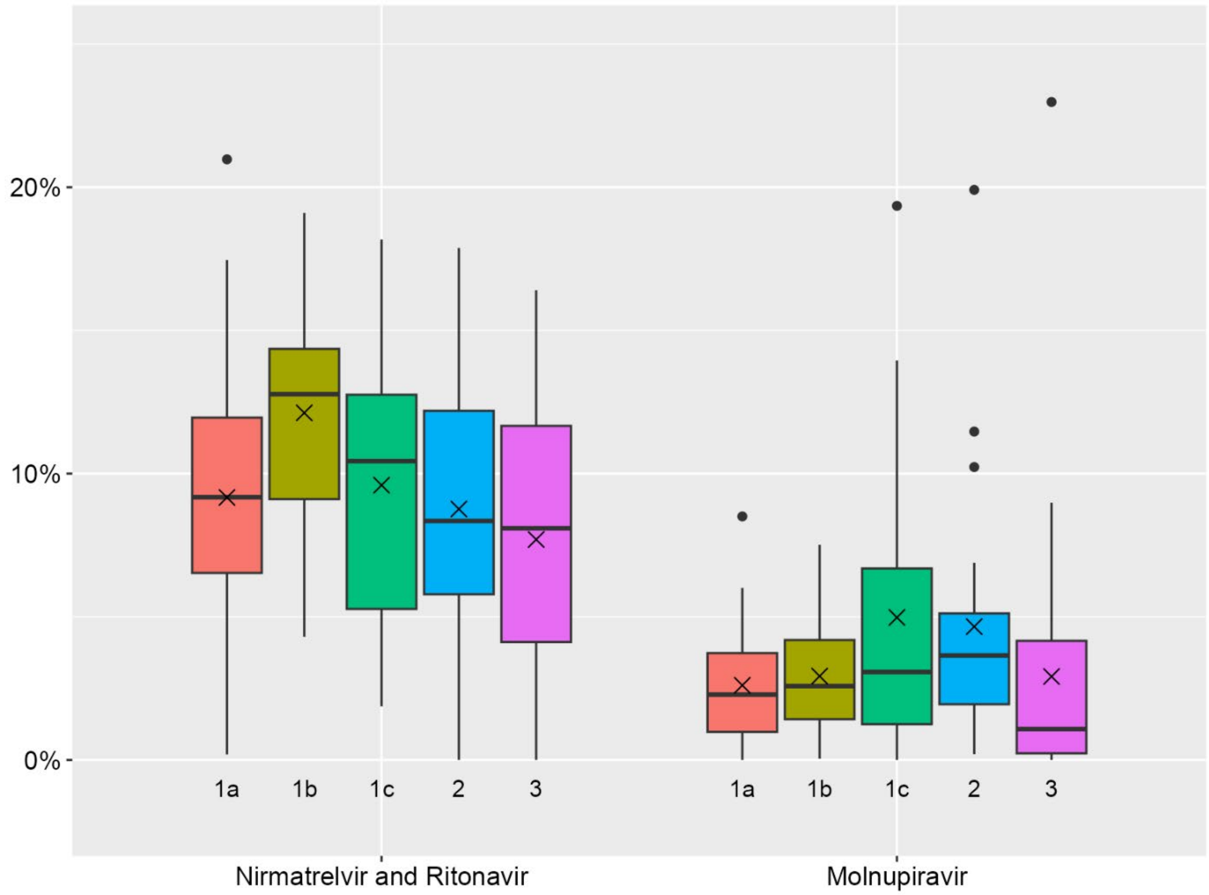

[a] Complexity levels include 1a, 1b, 1c, 2, or 3, with level 1a facilities being the most complex and level 3 facilities being the least complex. 1a-High Complexity facilities are facilities with high-volume, high-risk patients, most complex clinical programs, and large research and teaching programs). https://www.va.gov/oig/pubs/statements/VAOIG-statement-20190620-missal.pdf

C. Ratio of nirmatrelvir-ritonavir to molnupiravir at each facility[b]

© 2023 Yan L et al. *JAMA Network Open.*

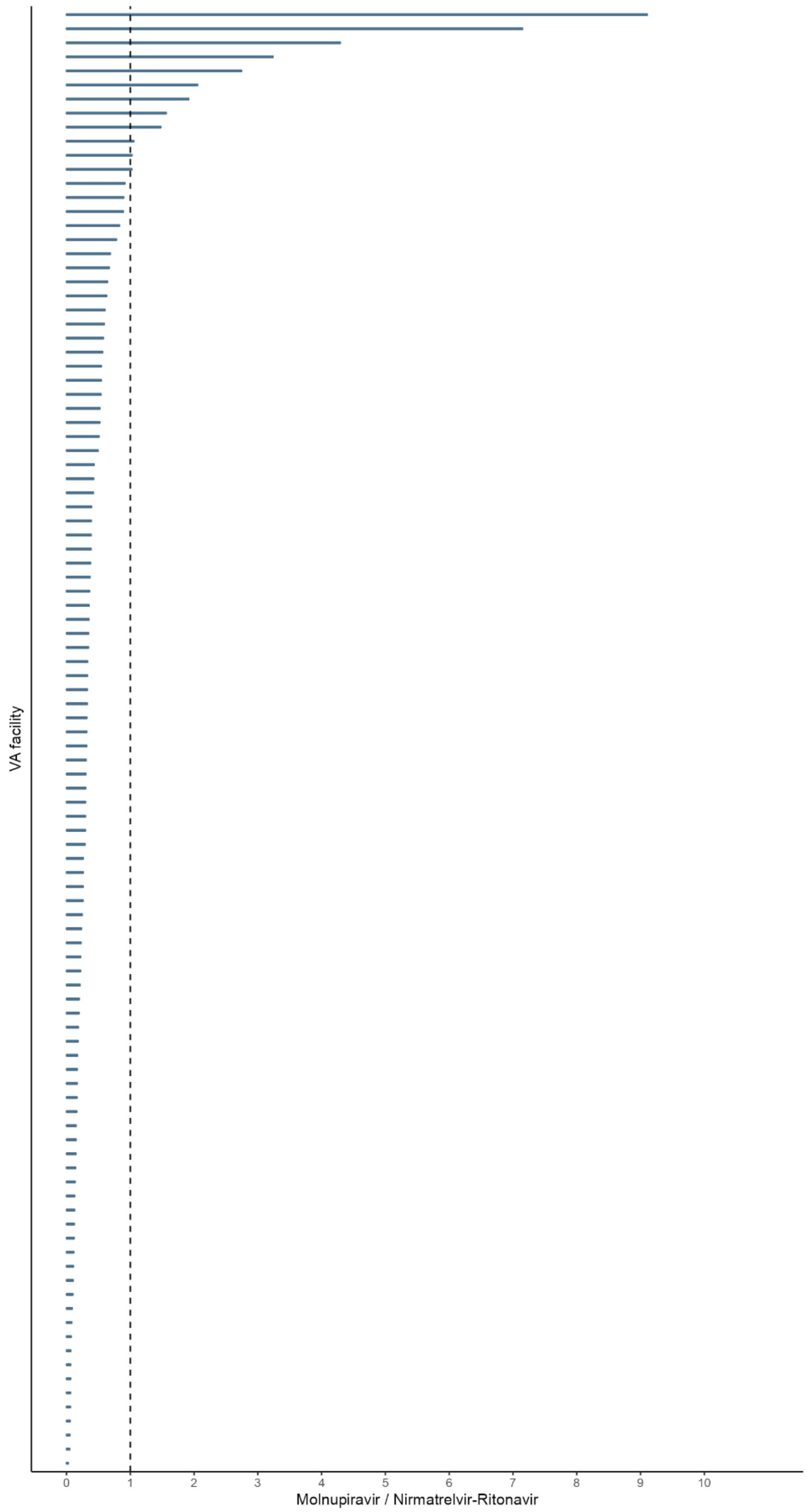

[b] Facilities with ≤10 courses of nirmatrelvir-ritonavir or molnupiravir were omitted to comply with CMS cell size suppression policy (https://resdac.org/articles/cms-cell-size-suppression-policy)

© 2023 Yan L et al. *JAMA Network Open.*
