# Supplementary material for: Anti–SARS-CoV-2 Pharmacotherapies Among Nonhospitalized US Veterans, January 2022 to January 2023
Source: JAMA Netw Open. 2023 Aug 31;6(8):e2331249. doi: 10.1001/jamanetworkopen.2023.31249 (PMC10472184; doi:10.1001/jamanetworkopen.2023.31249)
Supplement: Supplement 2. — Data Sharing Statement [file jamanetwopen-e2331249-s002.pdf]

Data Sharing Statement

Yan. Anti–SARS-CoV-2 Pharmacotherapies Among Nonhospitalized US Veterans, January 2022 to January 2023. *JAMA Netw Open*. Published online August 28, 2023. doi:10.1001/jamanetworkopen.2023.31249

Data
Data available: No
